# Short Telomere Syndrome presenting with pulmonary fibrosis, liver cirrhosis and hepatopulmonary syndrome: a case report

**DOI:** 10.1186/s12890-023-02378-8

**Published:** 2023-04-11

**Authors:** Andrew Baird, Marcio Gomes, Carolina A. Souza, Kate Magner, Gonzalo Alvarez

**Affiliations:** 1 Division of Respirology, Department of Medicine, University of Ottawa, The Ottawa Hospital, General Campus, 501 Smyth Road, Ottawa, ON K1H 8L6 Canada; 2 Ottawa Hospital Research Institute, Ottawa, ON Canada; 3 Department of Pathology, University of Ottawa, The Ottawa Hospital, Ottawa, ON Canada; 4 Department of Medical Imaging & Internal Medicine, Division of Respirology, University of Ottawa, The Ottawa Hospital, Ottawa, ON Canada

**Keywords:** Case report, Short teleomere syndrome, Pulmonary fibrosis

## Abstract

**Background:**

Idiopathic pulmonary fibrosis is thought to result from aberrant post-injury activation of epithelial cells leading to fibroblast proliferation and activation. A number of genetic aetiologies have been implicated in this disease process, including, among others, the short telomere syndromes. Short telomere syndromes follow an autosomal dominant pattern of inheritance resulting in shortened telomere length, which consequently leads to accelerated cell death. Organs with rapid cell turnover are most affected.

**Case presentation:**

We describe a case of a 53-year-old man with a chief complaint of cough and dyspnea on exertion. His presentation was otherwise significant for features of accelerated aging, including a history of osteoporosis and early greying, and a family history of pulmonary fibrosis in his father. Pulmonary function testing revealed a restrictive pattern with severely reduced diffusion capacity and high resolution CT of the chest showed diffuse lung disease with mild fibrosis, in pattern suggesting an alternative diagnosis to IPF. Biopsy of the lung was in keeping with chronic fibrosing interstitial pneumonia. Imaging of the abdomen showed splenomegaly, hepatic cirrhosis and portal hypertension. Transthoracic contrast echocardiogram showed intrapulmonary shunting consistent with hepatopulmonary syndrome. Given the constellation of early aging, idiopathic pulmonary fibrosis, cryptogenic cirrhosis and a family history of pulmonary fibrosis in this patient, the Short Telomere Syndrome was suspected. Peripheral blood was sent for Flow-cytometry FISH, which demonstrated granulocyte telomere length below the 10^th^ percentile for the patient’s age, consistent with a diagnosis of Short Telomere Syndrome in this clinical context. Targeted genetic testing of mutations known to be associated with short telomere was negative though it was acknowledged that the full spectrum of disease-causing mutations remains unknown. Given the extensive fibrosis on biopsy and his progressive hypoxemia he was treated with mycophenolate and prednisone. Ultimately, he developed progressive respiratory failure and underwent double lung and concurrent liver transplant 18 months after the initial diagnosis was made.

**Conclusions:**

Short Telomere Syndrome is a rare cause of end stage organ disease and testing lacks sensitivity making diagnosis challenging. Organ transplant is still the mainstay of treatment. Nevertheless, disease identification is important because of implications for family member screening and the possibility of future treatment options.

**Supplementary Information:**

The online version contains supplementary material available at 10.1186/s12890-023-02378-8.

## Background

Idiopathic pulmonary fibrosis is a common, progressive condition resulting in parenchymal scarring, lung function loss and debilitating symptoms. It is associated with a poor prognosis with median survival of approximately 3 years. Until recently, few, if any treatment options existed, with lung transplant remaining the primary definitve therapy for those that were appropriate candidates. In recent years, antifibrotic therapy has become the standard of care for those with IPF; however, the prognosis remains poor. It is currently thought that fibrosis in these patients is the result of aberrant post-injury activation of epithelial cells leading to fibroblast proliferation and activation [1]. In attempting to understand this disease process, and with the observation that a familial pattern is sometimes seen in patients with IPF, a number of genetic etiologies have been identified. These include genes resulting in a loss of telomere length maintenance (the so called short telomere syndromes (STS)) as well as familial pulmonary fibrosis (FPF), with numerous known genetic mutations in surfactant proteins and mucin 5B, among others [2].

## Case presentation

A 53-year-old male presented with a 3-year history of progressive cough and dyspnea on exertion. His past medical history included osteoporosis, gastroesopageal reflux, moderate obstructive sleep apnea and dyslipidemia. Physical exam was notable for grey hair, which had started in the fourth decade of life, and clubbing. No systemic features of connective tissue disease were noted. Family history was notable for pulmonary fibrosis in the patient’s father, who passed away in his seventh decade with autopsy findings suggestive of acute interstitial pneumonitis. The patient is a life-time non-smoker with no relevant exposures.

**Fig. 1:**
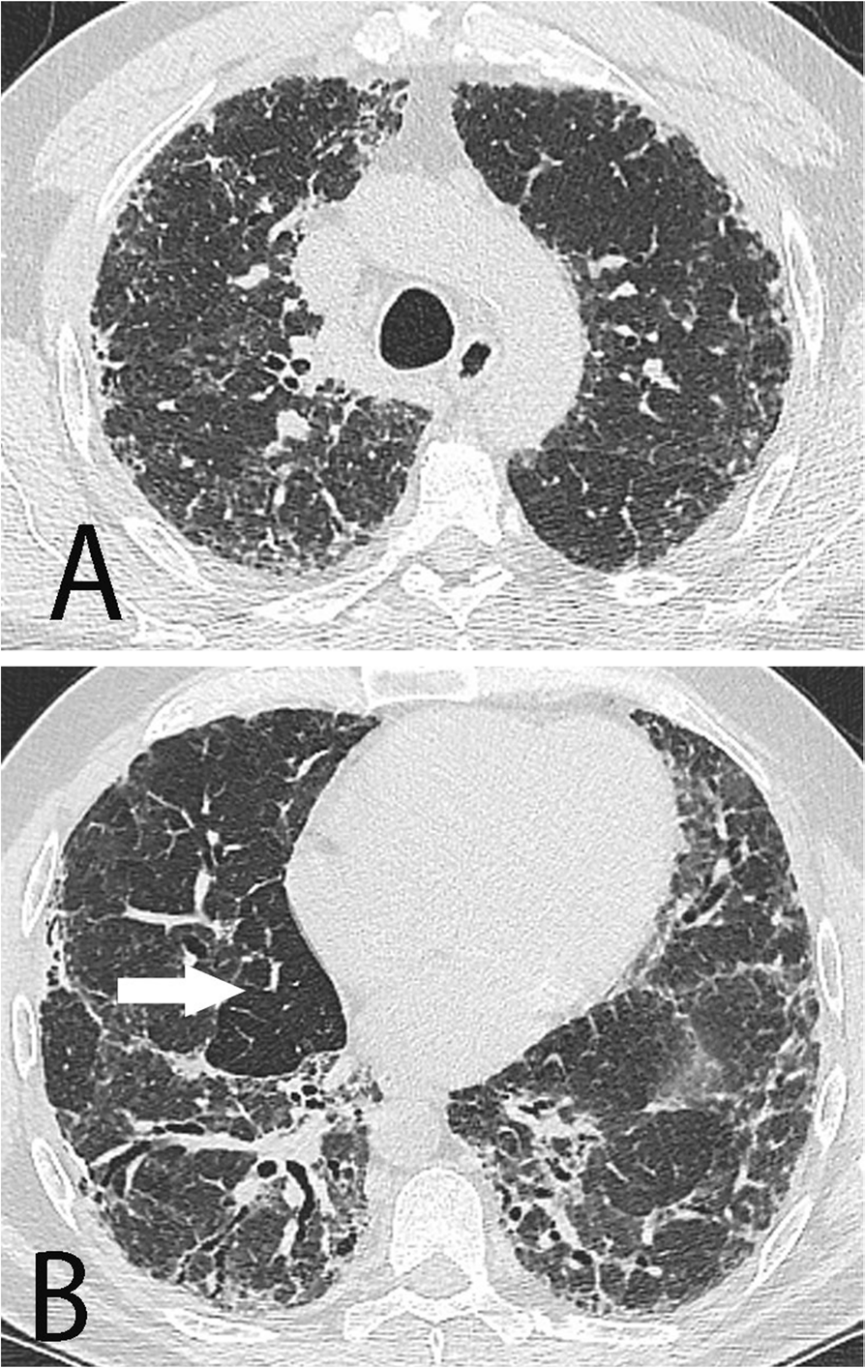
HRCT of the chest in the axial plane shows reticulation and traction bronchiectasis consistent with findings of moderate-severity interstitial fibrosis. There is no honeycombing. Patchy ground-glass opacities are present along with mosaic perfusion pattern with lobular areas of air-trapping (arrow, image B). The disease involve all pulmonary lobes, including upper lung zones (image A) but is more severe in the lower lobes (image B)

Pertinent investigations included pulmonary function tests demonstrating a restrictive process with severe reduction in diffusion capacity. High resolution computed tomography (HRCT) of the chest demonstrated an interstitial lung disease with moderate-severity fibrosis in keeping with an alternative diagnosis to IPF, according to current guidelines (Fig. 1). The overall appearance and distribution of the disease, notably the presence of ground-glass opacities and mosaic perfusion with air-trapping was suggestive of fibrotic hypersensitivity pneumonitis. Imaging of the abdomen demonstrated splenomegaly and a nodular liver compatible with hepatic cirrhosis. Isolated thrombocytopenia was also identified. Connective tissue disease work up revealed an elevated ANA 1:160 in a homogenous pattern, and was otherwise unremarkable.

**Fig. 2:**
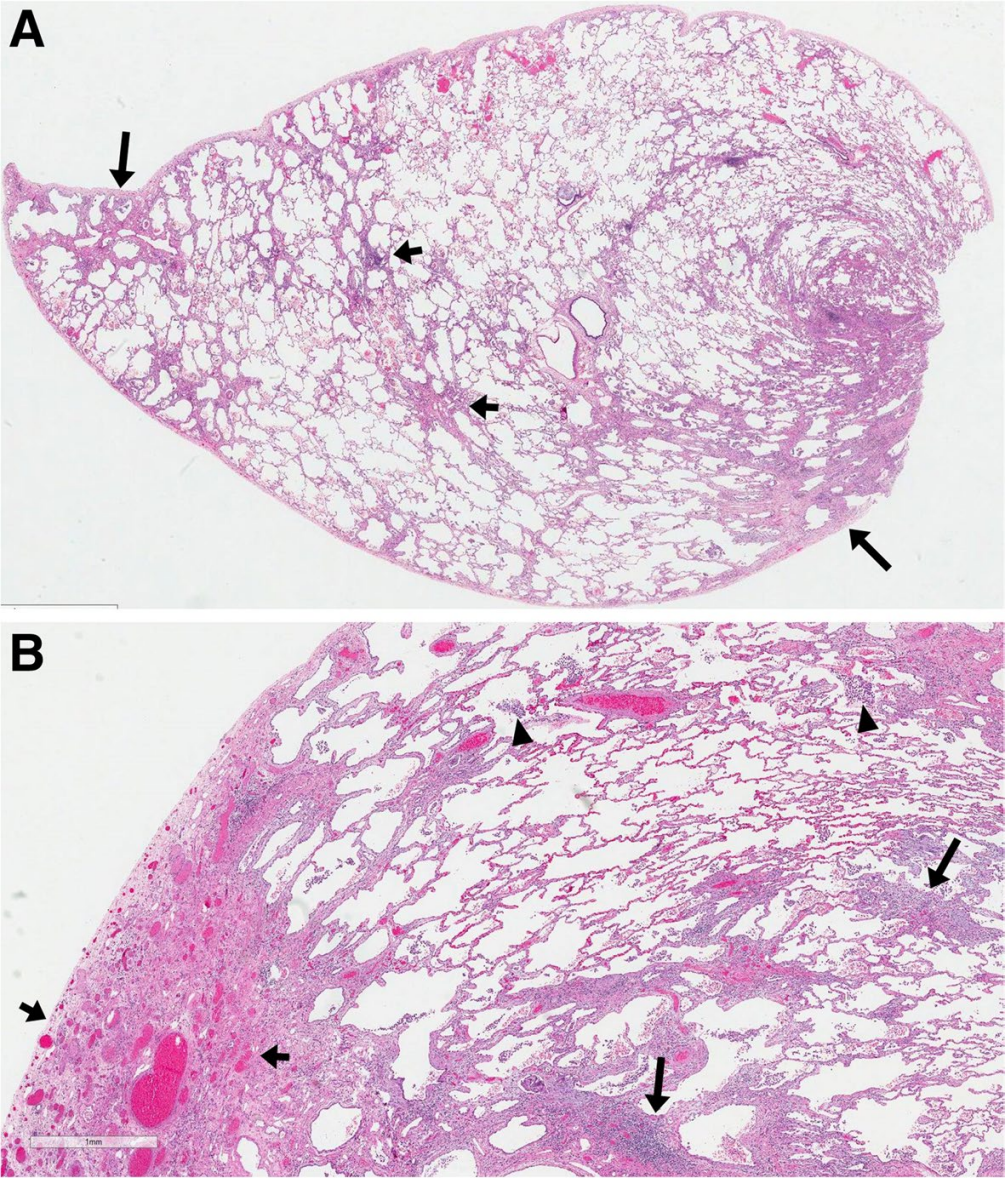
**a** Scanning magnification of H&E-stained slide of the upper lobe wedge lung biopsy showing patchy fibroinflammatory changes involving the centre (short arrows) and the periphery (long arrows) of the lobule. At higher magnification, areas of active inflammation and organizing fibrosis were also present (not shown). **b** Scanning magnification of H&E stained slide of the middle lobe wedge lung biopsy showing patchy fibro-inflammatory changes with predominant subpleural interstitial fibrosis and venous congestion and ectasia (short arrows). A moderate lymphocytic infiltrate is noted in the centrilobular region (long arrows), as well as intra-alveolar aggregates of hemosiderin-laden macrophages (arrowheds) indicative of alveolar hemorrhage. These vascular changes could be associated with the hepatopulmonary syndrome

After multidisciplinary review, the interstitial lung disease (ILD) was considered to be consistent with an alternative diagnosis to IPF, due to clinical and radiological discordance, and a surgical lung biopsy was performed (Fig. 2). Histologically, there was chronic active pneumonitis with extensive architectural remodelling and areas of microscopic honeycombing, reminiscent of usual interstitial pneumonia (UIP) pattern; however, the presence of more widespread active disease, prominent centrilobular involvement, moderate inflammatory infiltrate away from areas of remodelling, and patchy alveolar hemorrhage were not in keeping with UIP. The pattern was interpreted as indeterminate for UIP.

A subsequent liver ultrasound demonstrated evidence of cirrhosis with splenomegaly and portal hypertension. The patient had significant desaturation on ambulation out of keeping with the degree of fibrosis seen on imaging; therefore, a Transthoracic Contrast Echocardiogram was done (Additional file 1). It demonstrated clear intrapulmonary shunting which was consistent with a diagnosis of Hepatopulmonary Syndrome (HPS).

Given the presence of significant inflammation in the biopsy and worsening hypoxemia, the patient was started on Mycophenolate 500 mg BID and Prednisone 10 mg daily resulting in stability in his lung function. He was subsequently referred for both lung and liver transplant assessment.

In light of the family history of pulmonary fibrosis and clinical features of accelerated aging including early greying, osteoporosis, cirrhosis and pulmonary fibrosis, we suspected this patient may have a genetic predisposition to pulmonary fibrosis secondary to STS. Peripheral blood was sent for Flow-cytometry FISH which demonstrated granulocyte telomere length below the 10^th^ percentile for the patient’s age, consistent with a diagnosis of STS in this clinical context. A medical genetics consult was obtained and a next generation gene sequencing panel for ILD containing several autosomal dominant genes that cause short telomere syndrome (including SFTPA1, SFTPA2, TERT, TERC, TINF2, RTEL1, PARN, NAF1) was sent. While testing of pathogenic variants in these 26 genes known to be associated with interstitial lung disease was negative, it was also ackownledged that the full spectrum of mutations resulting in this disease are largely unknown at this time.

The patient ultimately developed progressive respiratory failure and underwent double lung and concurrent liver transplant 18 months after the initial diagnosis was made.

## Discussion and conclusions

STS represent a spectrum of inherited genetic disorders that follow an autosomal dominant pattern of inheritance resulting in shortened telomere length. Telomeres consist of nucleotide tandem repeats that serve to protect the ends of chromosomes during cell turnover and are often referred to as a “molecular clock”. Errors in the maintainance of telomere length can occur through defects in a number of known genes. Shortened telomeres lead to accelerated cell death and organ dysfunction, and organs with rapid cell turnover are primarily affected [2].

**Fig. 3:**
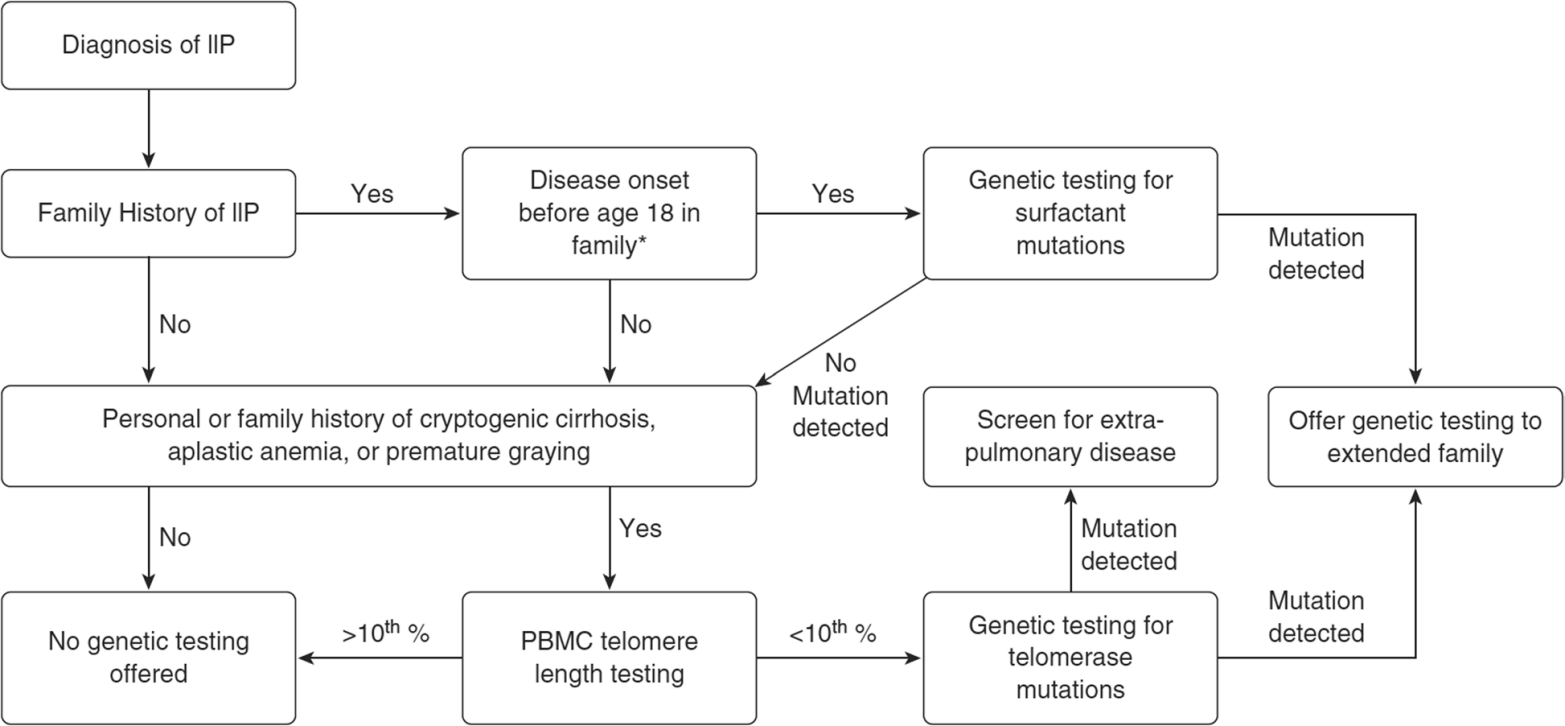
Short teleomere syndrome diagnostic algorithm proposed by Kopski et al.

Due to its rarity and limited awareness by clinicians, STS is likely underdiagnosed and there are no current guidelines to recommend a standard approach to diagnosis and treatment. The diagnostic approach proposed by Kropski et al. is appealing because of its simplicity (Fig. 3) [3]. In general, STS should be suspected when clinical clues exist, including premature greying of hair, IPF, bone marrow failure, cryptogenic cirrhosis, nodular regenerative hyperplasia or significant family history of these clinical findings. In patients less than 18 years of age with a family history and suggestive clinical features it is reasonable to proceed with evaluation for telomerase mutations. In older patients with more than one additional clinical feature of premature greying, cryptogenic cirrhosis or aplastic anemia, it is reasonable to first measure telomere length using flow-FISH [4], and in those with a length measurement less than the 10^th^ percentile, procede with further genetic testing. Importantly, the sensitivity of telomere length measurement declines with increasing age, making it more difficult to confirm the diagnosis in older patients, as was the case with our patient presented here [5].

Although the only currently available treatment for patients with STS remains organ transplant, androgens have been used with some success in patients with aplastic anemia [6]. Disease identification remains important due to the common autosomal dominant pattern of inheritance, which may lead to screening of offspring. Furthermore, patient’s with STS have increased incidence of important adverse effects including acute kidney injury, thrombocytopenia and bone marrow failure after lung transplant [7]. As new information has recently become available regarding the use of antifibrotic therapy in patient with non-IPF pulmonary fibrosis with similar efficacy, we suspect there may be a role for these therapies in STS but no clinical trials yet exist in this patient population [8].

In summary, we have described an interesting case of a patient with a constellation of clinical factors seen in STS, including early greying, osteoporosis, unclassifiable ILD, cryptogenic cirrhosis and HPS. This case highlights the challenges in genetic testing given the poor sensitivity, in addition to potential issues with telomere length measurement in older adults. Once more definitive treatment options exist, improvements in awareness, recognition and diagnosis of this interesting inheritable disorder will become critical.

## Supplementary Information

**Additional file 1.** Transthoracic contrast echocardiogram demonstrating significant shunting of contrast suggestive of heptopulmonary syndrome.

## Data Availability

The data generated during and/or analysed during the current study are available from the corresponding author on reasonable request.

## Abbreviations

STS: Short telomere syndrome
FPF: Familial pulmonary fibrosis
ILD: Interstitial lung disease
IPF: Idiopathic pulmonary fibrosis
UIP: Usual interstitial pneumonia
HPS: Hepatopulmonary syndrome
